# Epidural Stimulation Combined with Triple Gene Therapy for Spinal Cord Injury Treatment

**DOI:** 10.3390/ijms21238896

**Published:** 2020-11-24

**Authors:** Rustem Islamov, Farid Bashirov, Filip Fadeev, Roman Shevchenko, Andrei Izmailov, Vage Markosyan, Mikhail Sokolov, Maksim Kuznetsov, Maria Davleeva, Ravil Garifulin, Ilnur Salafutdinov, Leniz Nurullin, Yuriy Chelyshev, Igor Lavrov

**Affiliations:** 1 Kazan State Medical University, 420012 Kazan, Russia; faridbashirov@yandex.ru (F.B.); philip.fadeyev@gmail.com (F.F.); shev42006@yandex.ru (R.S.); andrei.izmaylov@kazangmu.ru (A.I.); vage.markosyan@gmail.com (V.M.); supermihon@yandex.ru (M.S.); qmaxksmu@yandex.ru (M.K.); izmailova_maria@mail.ru (M.D.); ravil.l16rus@mail.ru (R.G.); chelyshev-kzn@yandex.ru (Y.C.); 2 Institute of Fundamental Medicine and Biology, Kazan [Volga Region] Federal University, 420008 Kazan, Russia; sal.ilnur@gmail.com; 3 Kazan Institute of Biochemistry and Biophysics, Federal Research Center of Kazan Scientific Center of Russian Academy of Sciences, 420111 Kazan, Russia; leniz2001@mail.ru; 4 Department of Neurologic Surgery, Department of Biomedical Engineering, Mayo Clinic, Rochester, MN 55905, USA

**Keywords:** spinal cord injury, pigs, epidural electrical stimulation, cell-mediated gene therapy, human umbilical cord blood mononuclear cell, adenoviral vector, vascular endothelial growth factor, glial cell-derived neurotrophic factor, neural cell adhesion molecule

## Abstract

The translation of new therapies for spinal cord injury to clinical trials can be facilitated with large animal models close in morpho-physiological scale to humans. Here, we report functional restoration and morphological reorganization after spinal contusion in pigs, following a combined treatment of locomotor training facilitated with epidural electrical stimulation (EES) and cell-mediated triple gene therapy with umbilical cord blood mononuclear cells overexpressing recombinant vascular endothelial growth factor, glial-derived neurotrophic factor, and neural cell adhesion molecule. Preliminary results obtained on a small sample of pigs 2 months after spinal contusion revealed the difference in post-traumatic spinal cord outcomes in control and treated animals. In treated pigs, motor performance was enabled by EES and the corresponding morpho-functional changes in hind limb skeletal muscles were accompanied by the reorganization of the glial cell, the reaction of stress cell, and synaptic proteins. Our data demonstrate effects of combined EES-facilitated motor training and cell-mediated triple gene therapy after spinal contusion in large animals, informing a background for further animal studies and clinical translation.

## 1. Introduction

Spinal cord injury (SCI) is a complex condition with multiple cascades of changes such as cell death, inflammatory response, demyelination, as well as impairment of neural connectivity and functions. The successful treatment of the consequences of SCI requires an integrated therapeutic approach [1,2] with precise selection of the therapies to target specific mechanisms of pathological cascades in optimal combination. To achieve these goals, integrated technologies such as cell and gene therapy [3,4], electrical stimulation [5,6,7], motor exercises [8], selective blocking of the inhibitory molecules (Rho-ROCK inhibition) [9], electrochemical neuromodulation therapy [10,11,12,13], etc. have to be tested alone and in various combinations. Gene therapy and neurotrophic factors are generally considered to be promising for the stimulation of post-traumatic spinal cord recovery [14]. Several studies reported that the simultaneous delivery of several therapeutic transgenes encoding neurotrophic molecules, targeting different cell types and cell functions, may result in effective neuroregeneration [3]. The commonly performed direct (in vivo) or cell-mediated (ex vivo) delivery of transgenes encoding neurotrophic factors efficiently promotes the regeneration of damaged neurons, the growth and myelination of their processes, and restoration of the lost connections [3,4]. In previous studies, we confirmed the positive effect of a combination of transgenes encoding vascular endothelial growth factor (VEGF), glial cell neurotrophic factor (GDNF), and nerve cell adhesion molecule (NCAM) on the morpho-functional restoration of the spinal cord after contusion injury in rats [15,16] and pigs [17] models. 

VEGF, initially described as a vascular permeability factor with specific mitogenic activity for endothelial cells [18], has a known direct neurotrophic and neuroprotective impact on glial and neuronal cells within the CNS and PNS [19]. In neurogenesis, VEGF is involved in axon growth, synaptogenesis, and synaptic plasticity [20]. An important role of VEGF is shown in the pathogenesis and treatment of motor neuron diseases such as amyotrophic lateral sclerosis [21]. The local delivery of recombinant VEGF [22] or adenovirally delivered cDNA encoding VEGF in rats with the SCI model inhibit the entry of nerve cells into apoptosis [23]. Retrovirally transduced to overexpress VEGF, neural stem cells enhance the proliferation of glial progenitors, angiogenesis, and tissue sparing in rats after SCI [24].

GDNF signaling controls neuronal survival, axon guidance, and synapse formation in the developing and regenerating nervous system [25]. An intraspinal injection of adenoviral vector carrying cDNA encoding GDNF into the injured spinal cord had a positive effect on functional recovery in rats with SCI [26]. Recently, in our study of Schwann cells producing recombinant GDNF were used in a bioengineered scaffold to promote axons regeneration after a complete SCI [27].

NCAM plays a critical role in neurogenesis. In addition to interacting with each other and to the extracellular matrix (ECM) proteins, NCAM interacts with ion channels and cytokine and neurotransmitter receptors. NCAM is one of the key regulators of synapse formation, activity, and plasticity [28]. NCAM may as well activate tyrosine kinase receptors for fibroblast growth factor (FGF), epidermal growth factor (EGF), and nerve growth factor (NGF) [29]. Earlier, we suggested that the transduction of UCBMCs with an adenoviral vector carrying NCAM cDNA may not only have a positive effect on neuroregeneration, but also may enhance homing and survivability of the gene-modified UCBC after transplantation into ALS mice [30,31]. In the study, to enhance the therapeutic potential of UCBMC, we suggested using genetically engineered UCBMC for tandem delivery of neurotrophic factors (VEGF and GDNF) in combination with a neuronal cell adhesion molecule (NCAM).

**Figure 1:**
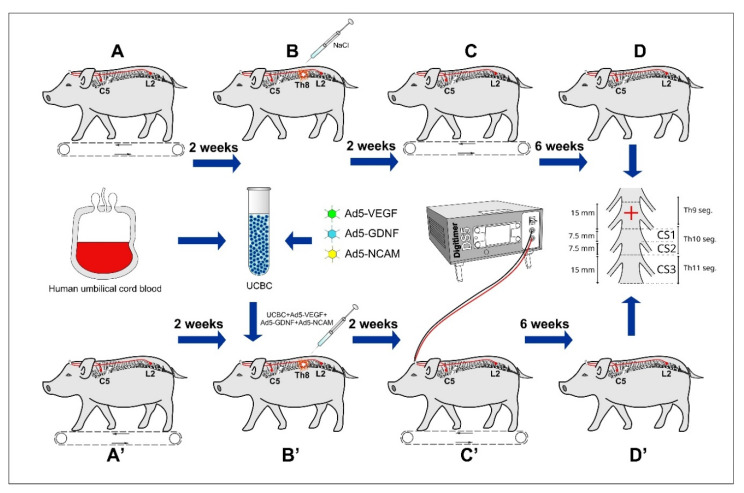
Study design. Upper panel: (**A**) Implantation of stimulating electrodes followed by recovery and training of animals to walk on a treadmill. (**B**) Spinal contusion followed by intrathecal administration of saline (0.9% NaCl) 4 h after injury. (**C**) Training on a treadmill every 2nd day started 2 weeks after injury. (**D**) Termination of the experiment at 60 days after injury. The caudal part of the spinal cord relative to the epicenter of injury (red cross) was divided into three segments (CS1, CS2, and CS3) for histological and molecular studies. Low panel: (**A**’) Implantation of stimulating electrodes followed by recovery and training of animals to walk on a treadmill. (**B**’) Spinal contusion followed by intrathecal administration of gene modified umbilical cord blood mononuclear cells (UCBC) 4 h after injury. (**C**’) Epidural electrical stimulation (EES) every 2nd day combined with training on a treadmill started 2 weeks after injury. (**D**’) Termination of the experiment at 60 days after injury and harvesting of the spinal cords for investigation. Intact healthy animals (not shown) were used to collect basic behavioral, electrophysiological, and histological data for comparative analysis.

Current preclinical studies and clinical trials demonstrate a positive effect of epidural electrical stimulation (EES) on functional improvement and morphological reorganization after SCI [6,7,32,33,34,35]. This effect was related to the facilitation of a spinal cord neural network called “central pattern generator”, enabling the locomotor function after SCI [36,37,38], as well as the activation of various signaling intracellular cascades [39], autonomic and vascular mechanisms [40], and integrating multiple sensory systems [7]. The emergent studies clearly indicate the benefits of EES or gene therapy, applied alone, raising a critical question about the therapeutic effect of a combination of neuromodulations by EES and neuroprotection with recombinant neurotrophic factors on animals and eventually in humans. Recently, we demonstrated that the EES applied above and below the injury, combined with intrathecal administration of gene modified UCBC overexpressing recombinant VEGF, GDNF, and NCAM, has a cumulative positive effect on remodeling of the spinal cord and promotes locomotor recovery in rodents with SCI [41]. However, the positive results obtained in the experiments on small animals (rodents) cannot be directly translated to humans. To facilitate this translation to the clinical studies and further expand our understanding of the cellular and molecular mechanisms of SCI on large animals closer to humans in morpho-physiological scale, we selected a pig model of SCI with close to human spinal cord functional neuroanatomy, physiological, and biochemical characteristics [33,42,43,44,45]. In the present pilot study on the limited number of animals, we demonstrate for the first time the effect of a combination of EES and ex vivo triple gene therapy applied following SCI in pigs (Figure 1).

## 2. Results

### 2.1. Functional Restoration

**Figure 2:**
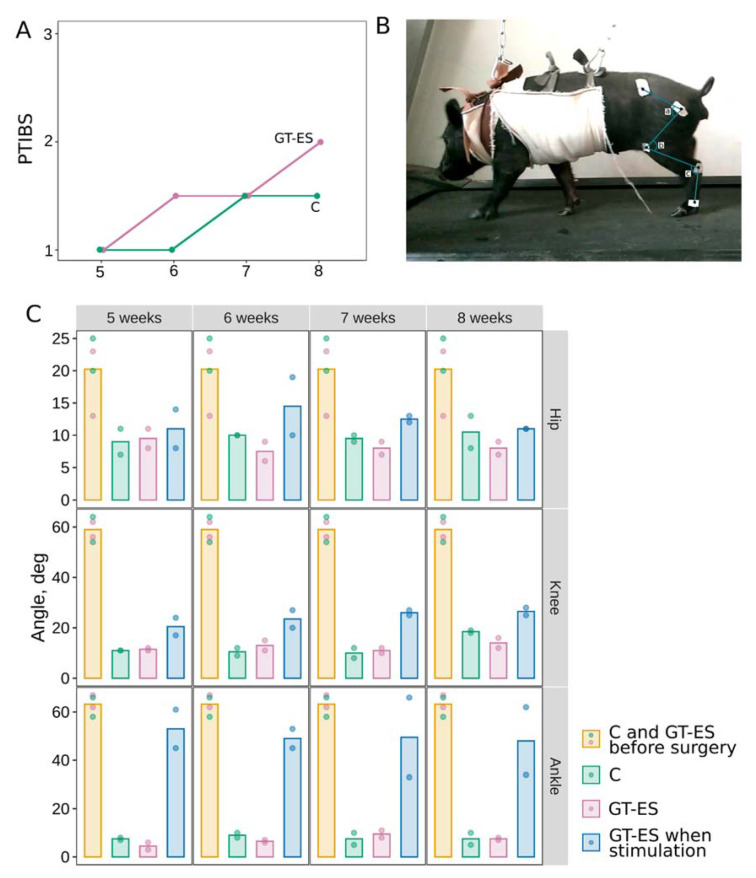
Evaluation of the motor recovery. (**A**) Porcine thoracic injury behavioral scale (PTIBS) scores in control (green) and treated (pink) groups. (**B**) Video recording of the hip (**A**), knee (**B**), and ankle (**C**) joint kinematics during stepping on a treadmill was performed in intact healthy pigs 1 week before surgery and at different time points after spinal cord contusion injury. (**C**) The angle (degree) of movements measured in the hip, knee, and ankle joints in experimental pigs before surgery (I, yellow bar) and week 5, 6, 7, and 8 after spinal cord injury (SCI) in control (C, green bar) and treated (GT-ES, pink bar) groups during training on the treadmill. The blue bar (GT-ES when stimulation) represents data of animals from the GT-ES group during training on the treadmill enabled with epidural stimulation at the L2 segment. Average angles of five sequential step cycles for each animal are presented as color points; bars correspond to group-wise average values. The colors of points in I (yellow bars) correspond to the colors of C and GT-ES bars.

The behavioral examination was conducted by an assessment of active and passive movements of the fore- and hind limbs, pain sensitivity (skin pinch test), legs muscle tone, monitoring of urinal and fecal passage before surgery and subsequently, throughout the experiment. Four weeks after SCI, animals from both control (C) and therapeutic (GT-ES) groups were unable to stand and walk using their hind limbs. The neurological examination revealed a central type of paralysis of the hind limbs (loss of active movements and pain sensitivity, increased tone of the skeletal muscles, and reduction of the passive movements). Six weeks after SCI in the GT-ES group, the PTIBS score was increased. Moreover, during the skin pinch test of the hind limbs, treated pigs demonstrated a vocal and motor reaction. Eight weeks after surgery in the GT-ES group, a further increase of active movements reached 2 points on a 10-point PTIBS scale with the reestablishment of voluntary voiding (Figure 2A). Motor performances in untreated pigs (C group) by the end of the experiment reached only the 6-week level in animals from the treated (GT-ES) group.

The hip, knee, and ankle joints movements were decreased in both control and therapeutic groups at 5, 6, 7, and 8 weeks after SCI when compared to data obtained from intact pigs before surgery (Figure 2B,C). Movements performed without EES were highly reduced in the experimental groups compared to the intact group. The average angle of flexion in the hip joint in intact animals was 20.2 degrees at all stages of the study. In animals from the C and GT-ES groups, the average angle range was reduced to 9 and 9.5 after 5 weeks, to 10 and 7.5 after 6 weeks, to 9.5 and 8 after 7 weeks, and to 8 and 11 after 8 weeks, respectively. The average angle of flexion in the knee joint in intact animals was 59.0 degrees and was highly decreased at all time points in experimental groups. After 5 weeks, pigs in C and GT-ES groups demonstrated a reduced average angle range to 11 and 11.5, after 6 weeks to 10.5 and 13, after 7 weeks to 10 and 11, and after 8 weeks to 18.5 and 14, correspondingly. The average angle of flexion in the ankle joint in intact animals was 62.2 degrees. At all-time points in the experimental groups, the movement in the ankle joint was greatly reduced compared to the control animals. At week 5 in pigs from the C and GT-ES groups, the average angle range was reduced to 7.5 and 4.5, at 6 weeks to 9 and 6.5, at 7 weeks to 7.5 and 9.5, and at 8 weeks to 7.5 and 7.5, correspondingly. An analysis of the hip, knee, and ankle joints movements in GT-ES animals during EES at the L2 level did not reveal the changes in the hip joint and demonstrated an increase in the angle of movement in the knee and ankle joints. The hip angle in GT-ES pigs when stimulated did not show a noticeable difference from GT-ES pigs without stimulation and from control pigs and was 11, 14.5, 12.5, and 11 degrees in average at 5, 6, 7, and 8 weeks after SCI, correspondingly. During EES at the L2 spinal segment, GT-ES pigs demonstrated a substantial increase in the knee joint movement at week 5 (20.5) and week 6 (23.5) and further increased at week 7 (26) and week 8 (26.5), in contrast to GT-ES pigs without EES. The best motor outcome during EES was observed in GT-ES pigs in the ankle joint, which was increased at week 5 (53), week 6 (49), week 7 (49.5), and week 8 (48), in contrast to the same animals (GT-ES group) without EES and to the control animals, but was similar to the intact animals (Video S1).

**Figure 3:**
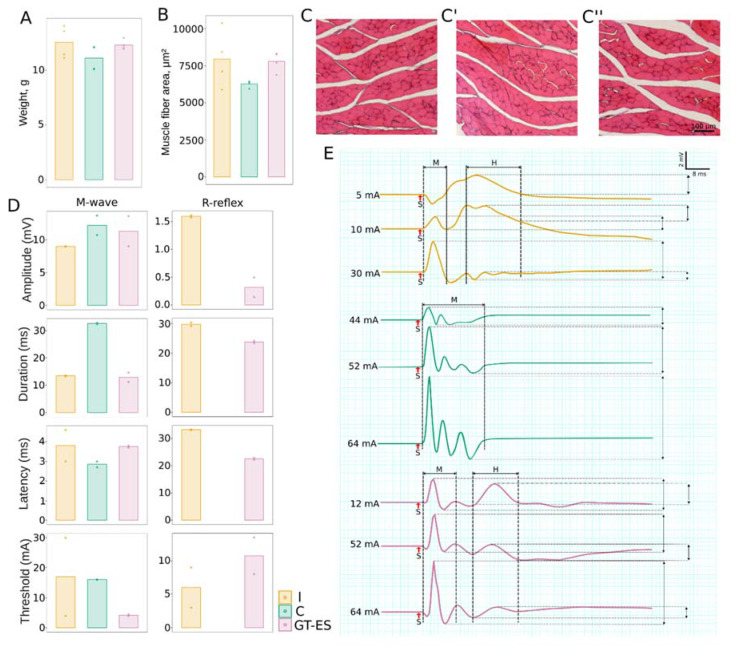
Assessment of hind limb skeletal muscles and an electrophysiological evaluation of M- and H-reflex. (**A**) Tibialis anterior muscles weighing analysis. (**B**) Evaluation of muscle fibers area in the tibialis anterior muscle. (**C**,**C**’,**C**’’) Cross sections of the tibialis anterior muscle stained with hematoxylin and eosin from I, C, and GT-ES groups, correspondingly. (**D**) Electrophysiological study of M- and H-reflex in the soleus muscle. (**E**) Parameters of the electromyography (EMG) signal. Data obtained for each animal are presented as points; bars represent group-wise average values.

An evaluation of the hind limb skeletal muscles at 60 days after SCI revealed that the average weight of m. tibialis anterior in the intact group was 11.85 and was similar to C (11.08) and GT-ES groups (12.27) (Figure 3A). A morphometric analysis of muscle fibers area (μm^2^) in the tibialis anterior muscle revealed that the average area of muscle fibers in pigs from the therapeutic group (7783 μm^2^) was higher compared to the control (6207 μm^2^) and was similar to the intact group (7871 μm^2^) (Figure 3B). The electromyography (EMG) of the soleus muscle at 60 days after SCI in control pigs revealed a polyphasic M-response and no H-reflex (Figure 3C). During an incremental increase in the intensity of sciatic nerve stimulation until the registration of the maximum amplitude of M response, the polyphasic shape of the M-response did not change. At the same time, in animals from the treated group, EMG revealed both the M-response and H-reflex.

Overall, the behavioral test, kinematics hind limb joints, electromyography, and analysis of the muscle fibers area of hind limb skeletal muscles, suggest that a combination of EES and ex vivo triple gene therapy may potentially be associated with the higher rate of locomotor performance in treated pigs. However, this still requires further study with more animals for claiming an effect of the proposed intervention.

### 2.2. Spinal Cord Remodeling

**Figure 4:**
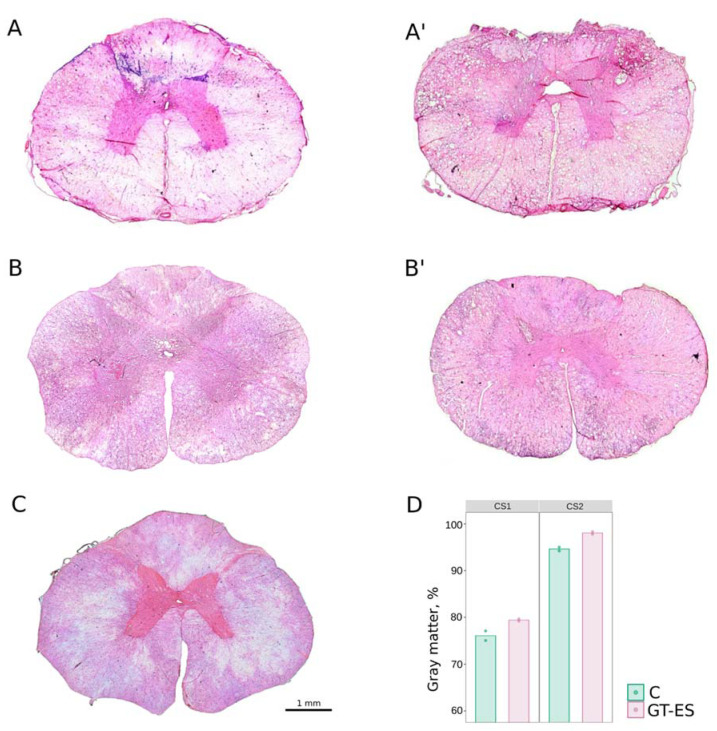
The area of the preserved gray matter in spinal cords at 60 days after SCI in CS1 and CS2 segments. (**A**,**A**’) Cross sections of spinal cords stained with hematoxylin and eosin in control (C) and treated (GT-ES) animals from CS1. (**B**,**B**’) Spinal cords of control and treated pigs from CS2. (**C**) An intact spinal cord. (**D**) The relative area of preserved gray matter in control (C) and treated (GT-ES) pigs. Values averaged within animals are presented as points; bars represent group-wise average values.

The morphometric analysis of the spinal cord gray matter in CS1 and CS2 segments in control and therapeutic groups at 60 days after SCI demonstrated a more preserved nervous tissue area in both groups in CS2 segments (farther from the epicenter of injury compared to CS1) (Figure 4). The sparing tissue in CS1 and CS2 segments that converted into percent values were lower in the control (C) (75.9 and 94.61) compared to the GT-ES group (79.36 and 98.1) (Figure 4B).

**Figure 5:**
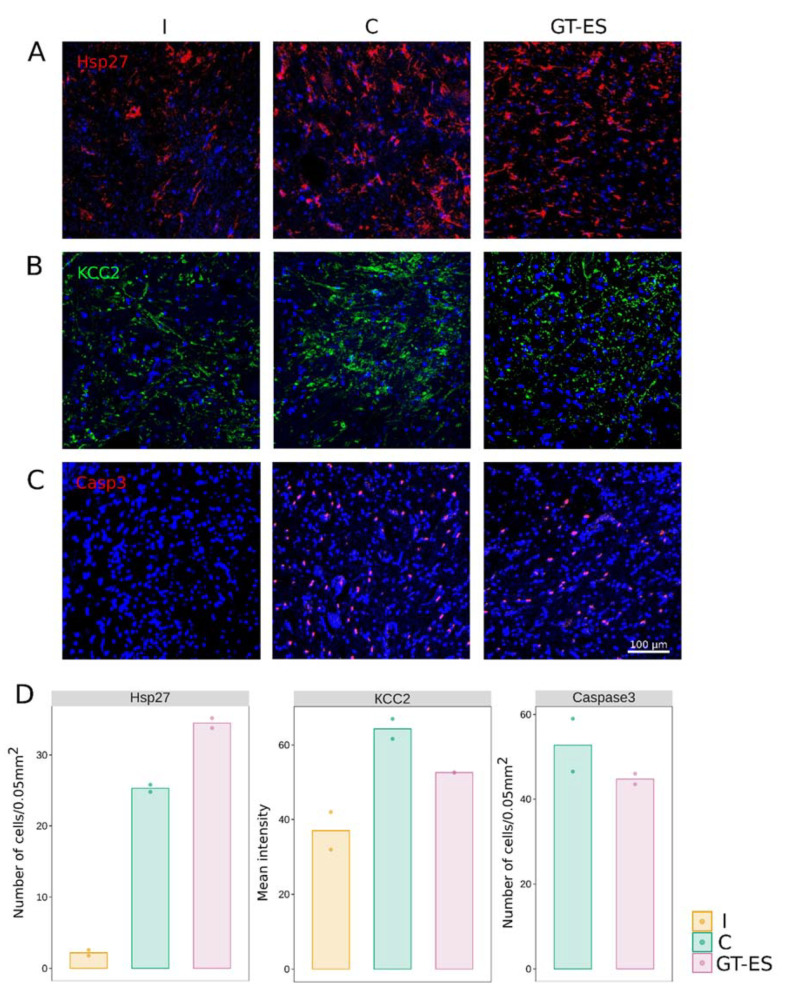
Immunofluorescent analysis of cell stress molecules in spinal cord ventral horns at 60 days after SCI in the CS2 segment. (**A**) Immunoexpression of a heat shock protein of 27 kDa (Hsp27). (**B**) Immunoexpression of a neuron specific K^+^-Cl^−^ co-transporter (KCC2). (**C**) Immunoexpression of a pro-apoptotic protein Caspase3. Nuclei are counterstained with DAPI (blue). (**D**) KCC2 mean intensity and the number of Hsp27 and cCaspase3-positive cells in intact (I), control (C), and treated (GT-ES) pigs. Caspase3-positive cells in the intact (I) group were not observed. Values averaged within animals are presented as points; bars represent group-wise average values.

The immunofluorescent study of the spinal cord at 60 days after SCI revealed Caspase3-positive cells in ventral horn (VH) in both C and GT-ES groups (Figure 5C,D). In intact spinal cords, Caspase3-positive cells were not found. The cell count showed an average decrease in the therapeutic group (44.8) compared to the control group (52.8). Meanwhile, the number of the Hsp27-positive cells in VH was higher in pigs from the therapeutic group (34.5) compared to control (25.3) and intact (2.2) groups (Figure 5A,D). The level of potassium-chloride cotransporter protein (KCC2) immunoexpression in VH was increased in control (64.2) and therapeutic (52.6) groups compared to the intact group (38.0). However, in treated pigs, the level of KCC2 was lower compared to control animals (Figure 5B,D).

**Figure 6:**
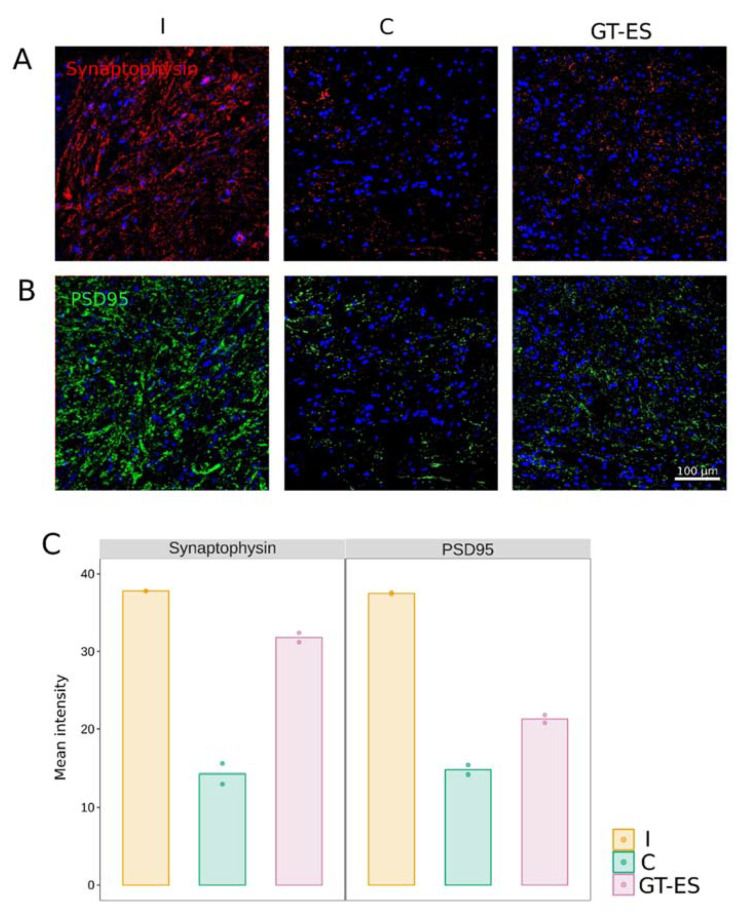
Immunoexpression of synaptic proteins in spinal cord ventral horns at 60 days after SCI. (**A**) Immunofluorescent staining with antibodies to Synaptophysin (synaptic vesicles protein). (**B**) Immunofluorescent staining with antibodies to postsynaptic density protein 95 kDa (PSD95). (**C**) Merged images representing Synaptophysin and PSD95. Nuclei are counterstained with DAPI (blue). Values averaged within animals are presented as points; bars represent group-wise average values.

The average fluorescence intensity of Synaptophysin was decreased in C (14.3) and GT-ES (31.8) experimental groups in comparison to intact animals (37.8). However, the higher level of Synaptophysin was found in treated compared to control animals (Figure 6A,D). The analysis of PSD95 immunoexpression demonstrated the same pattern of changes—decrease in control (14.8) and treated (21.3) pigs in comparison with the intact animals (37.5) and higher level of PSD95 in the GT-ES group compared to the C group (Figure 6B,D).

**Figure 7:**
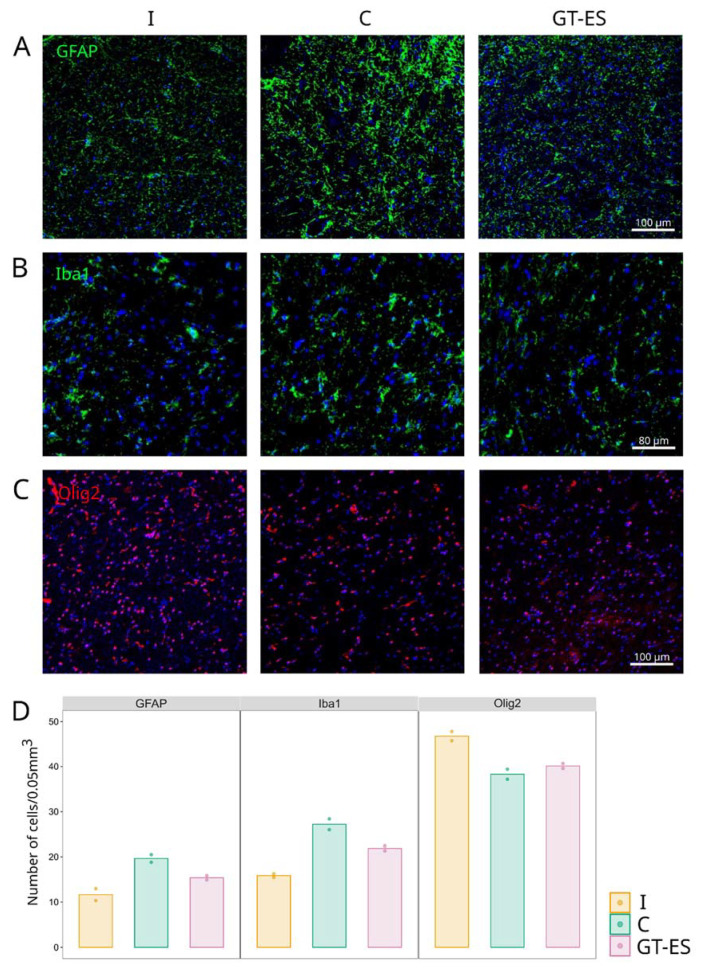
Immunofluorescent analysis of glial cells reorganization in spinal cord ventral horns at 60 days after SCI. (**A**) Astrocytes were visualized with antibodies against glial fibrilar acidic proteins (GFAP). (**B**) Microglial cells against ionized calcium binding adaptor molecule 1 (Iba1). (**C**) Oligodendroglial cell against transcription factor Olig2. Nuclei were counterstained with DAPI (blue). (**D**) Mean number of cells in intact (I), control (C), and treated (GT-ES) pigs. Values averaged within animals are presented as points; bars represent group-wise average values.

Neuroglial Olig2-positive cells (oligodendrocytes), GFAP-positive cells (astrocytes), and Iba1-positive cells (microglia) were counted in the VH of the spinal cord in intact and experimental pigs (Figure 7). The average number of GFAP-positive and Iba1-positive cells was higher in animals from control (19.73 and 27.09) and therapeutic (15.31 and 21.92) groups compared to the intact group (11.55 and 15.78), correspondingly. The average number of astrocytes and microglial cells were lower in the therapeutic group in comparison with control animals (Figure 7A,B,D). Counts of Olig2-positive cells were lower in both C (38.18) and GT-ES (40.08) groups when compared to the intact group (46.89) (Figure 7C,D). The cellular and molecular changes in the post-traumatic spinal cord indicate a specific effect of combined EES and triple gene therapy on the remodeling of the spinal cord in treated pigs.

### 2.3. Neurotransmission Gene Expression

**Figure 8:**
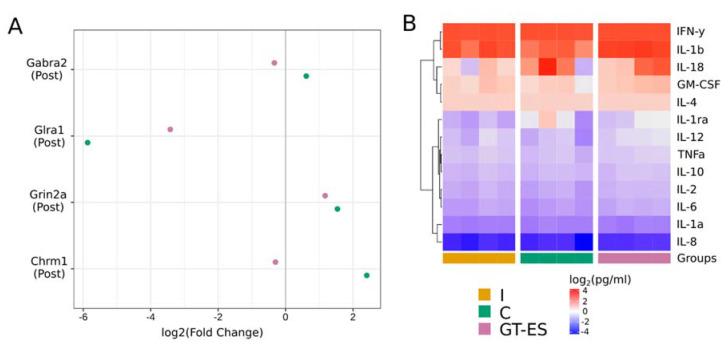
Cytokines profiling and synaptic gene expression in the spinal cord at 60 days after SCI. (**A**) Comparison of target genes expression. The results are represented as an average log2(fold change) in control (C, green points) and treated (GT-ES, pink points) pigs relative to the expression level in the intact group. Threshold cycles (Ct) were obtained for two animals (two repeats per animal) in each group and normalized by corresponding *Gapdh* values. (**B**) Heatmap representing a log-transformed cytokines absolute concentration in spinal cord samples, where each column represents tissue samples (two samples per animal). Columns are bunched by experimental groups (I, C, and GT-ES). Cytokines are ordered by the results of hierarchical agglomerative clustering.

Using RT-PCR assay, the expression of postsynaptic genes of cholinergic (*Chrm1*), glutamatergic (*Grin2a*), glycinergic (*Glra1*), and GABAergic (*Gabra2*) was investigated in the CS3 segment of spinal cords (Figure 8A). The level of *Chrm1* (muscarinic acetylcholine receptor M1) was increased (5-fold change) in control animals, indicating a higher level of the excitatory neurotransmission system. A substantial decrease in the expression of *Glra1* (glycine receptor subunit alpha-1) was found in both control (58.5-fold change) and therapeutic (10.7-fold change) groups, indicating a lower level of the inhibitory neurotransmission spinal system, although, in the therapeutic group, the decrease in *Glra1* expression was less pronounced compared to the control animals.

### 2.4. Inflammatory Response

Erythropoietin (EPO), granulocyte colony-stimulating factor (G-CSF), granulocyte-macrophage colony-stimulating factor (GM-CSF), interferon-beta (IFN-β), interferon-gamma (IFN-γ), interleukin-4 (IL-4), interleukin-10 (IL-10), and interleukin-12 (IL-12) are considered as cytokines with a neuroprotective potential. Meanwhile, chemokine (C-X-C Motif) ligand 10 (CXCL10), interleukin-1 (IL-1), interleukin-17 (IL-17), and tumor necrosis factor-alpha (TNF-α) may also cause the secondary death of neural cells [46]. In the present study, a multiplex cytokine analysis was employed to evaluate the inflammatory mediators’ profile in the CS3 segment of spinal cords at 60 days after SCI. This analysis did not reveal differences in the content of endogenous cytokines, chemokines, and growth factors in animals from the control and therapeutic groups when compared to the intact group (Figure 8B). Changes in the immune response were not detected in both experimental groups, indicating an extinction of the inflammatory reactions by that time.

## 3. Discussion

The limited regeneration after SCI is related to static neural cells population, significant axonal length, and specific properties of neuropile [47]. The destruction of the blood-brain barrier (BBB), development of microcirculation deficiency, and inflammatory processes also play a negative role in spinal cord recovery [48,49,50]. A shift to positive morphological and functional outcomes in post-traumatic spinal cord requires activation of endogenous regenerative potentials by employing various exogenous stimulants of neuroregeneration.

Previously, we have successfully tested the effect of a combined treatment with multisite EES and ex vivo triple gene therapy on the morpho-functional recovery of spinal cord in rats with SCI [41]. Four hours after a moderate spinal cord contusion, rats were intrathecally infused with gene modified UCBC overexpressing recombinant VEGF, GDNF, and NCAM and 3 days after injury, the rats were given EES simultaneously above the lesion site at C5 and below—at L2. In treated animals on the 30th day after SCI, we have observed improved knee joint movement, preserved hind limb skeletal muscle atrophy, and enhanced monosynaptic responses of spinal motoneurons, which were in line with higher sparing of the gray and white matter, higher level expression of heat shock and synaptic proteins, increased proliferation of oligodendroglial cells, and reduced astrogliosis in the post-traumatic spinal cord.

Similarly, in our present study, pigs with contusion injury at Th8-Th9 received 4 h later an intrathecal infusion of UCBC overexpressing recombinant VEGF, GDNF, and NCAM and 2 weeks after SCI received EES at C5 and L2 levels for 6 weeks. Stimulation at C5 was performed for the activation of the cervical network and propriospinal system together with stimulation of the axonal growth [51,52,53,54]. Stimulation at L2 was performed primarily for the activation of central pattern generators and enabling of stepping [6,55] for 4 weeks of EES-facilitated locomotor training. The behavioral, electrophysiological, histological, and molecular analyses revealed an improvement in animals treated with combined EES and triple gene therapy, compared to control. A decreased inflammatory response in the spinal cord was found in both control and treated animals. The morphometric analysis found that the volume of the pathological cavities was similarly higher in CS1 (closer to the epicenter contusion injury) and lower in CS2 (7.5 mm farther from CS1) both in control and treated animals. A similar number of Caspase3-positive cells in CS2 as well was found in pigs from C and GT-ES groups. Although we found an increase in the anti-stress protein Hsp27 immunoexpression in animals from the therapeutic group. Recent studies demonstrated that the Hsp27 expression was elevated in the ventral horn of the injured spinal cord and that the upregulation of Hsp27 can protect neurons from apoptosis after a lumbosacral nerve root avulsion [56]. The level of potassium-chloride cotransporter (KCC2) originally characterized as a regulator of cell volume was less prominent in treated pigs, compared to the control animals [57]. However, the immunoexpression of KCC2 in the GT-ES group was higher compared to the intact animals. This observation corresponds to the view that activity-based therapies and an increase in neurotrophic factors expression modulate KCC2 plasticity resulting in a decrease in nociceptive and spasticity circuits in the lumbar spinal cord [58,59]. The pattern of Hsp27 and KCC2 may be indicated on the regeneration processes in the spinal cord in treated pigs. The neuraglial cells reorganization in the spinal cord of pigs from the therapeutic group was due to a reduction of astrogliosis (a decrease in the number of GFAP-positive astrocytes and Iba1-positive microglia). The remodeling of the post-traumatic spinal cord and a higher level of presynaptic (Synaptophysin) and postsynaptic (PSD95) proteins expression in ventral horns in treated pigs were in line with the electrophysiological properties of hind limb skeletal muscles. The pattern of M- and H-responses of the soleus muscle and RT-PCR results of the expression of postsynaptic genes in the spinal cord suggest the reorganization of neuromotor units in treated animals. The expression of glycerinergic *Glra1* gene was found in interneurons in the dorsal horn of the spinal cord [60] and our finding of a decrease in the expression of *Glra1* in control pigs correspond to the results obtained in rodents with SCI [61]. Downregulation of *Glra1* and the absence of H-reflex in control animals may be considered as a response to the loss of inhibitory interneurons located in the dorsal horns—at the site with direct damage. In the study, results of RT-PCR and multiplex cytokine analysis were obtained from the CS3 segment. However, in a further investigation of molecular changes during neuroregeneration, the data precisely from dorsal and ventral horns at CS2 should be obtained.

The observed voluntary locomotor performance in PTIBS as well as the electrophysiological properties of the soleus muscle and reduction, compared to the control animals’ atrophy of m. tibialis anterior, may correspond to EES-enabled ankle joint movements in treated pigs. It is important to note that during the sessions of training and assessment, the motor performance was better in the presence of EES compared to the performance without EES, indicating a critical role of neuromodulation in the maintenance of a rhythmic activity in large animals even with combined gene and neuromodulation therapy. The effect of EES on propiospinal axons and interneurons distributed across multiple spinal segments may activate sprouting and interaction between different segments and between the corticospinal axons and propriospinal neurons. Therefore, this facilitates the formation of the translesional spinal neural network which may mediate and amplify interrupted by SCI signals [62,63]. In addition to the direct or indirect activation of neurons and the spinal cord network, electrical stimulations via voltage-sensitive channels can also result in some activation of the expression of genes encoding neurotrophic factors [64,65].

Recent clinical studies demonstrate that patients with a motor complete spinal cord injury (AIS-A and B) in the presence of EES were able to control volitional motion and independent walking, indicating a discomplete type of injury [34,66]. Other works demonstrated a significant improvement in the motor performance achieved with a non-invasive transcutaneous electrical stimulation [67,68]. Although current findings demonstrate a beneficial effect of both approaches, they may not be completely replicable and could involve different mechanisms. Therefore, compared to the epidural stimulation, which is effective in restoration of the volitional control of leg movement, transcutaneous stimulation appears to be less effective in the volitional control. Although, a better effect was demonstrated on the balance and posture control.

The human UCB cells are immunologically immature with low immunogenicity [69,70], which are widely used in preclinical studies in animals without immunosuppression therapy [71,72] and clinical trials in humans for the treatment of nonhematopoietic diseases [73,74]. A therapeutic efficacy of allogenic UCBC was shown after transplantation into patients with chronic SCI [73,75,76]. In our study, a multiplex cytokine analysis did not reveal differences in the level of pro-inflammatory cytokines and chemokines in animals from the GT-ES group when compared to the intact group. These data indicate the absence of an UCBC-induced systemic immune response. In addition, if a local cytotoxic reaction to these cells was found in the spinal cord, it is either weak or absent. The survivability of the intrathecally transplanted UCBC in rats was confirmed in our early investigations [15].

Most of the studies on small animal models and primary rodents demonstrated positive therapeutic effects of pharmacological, biomaterial, genetic, or neuromodulation therapies on functional recovery after SCI. Ideally, the effect in the rodent’s treatment strategies has to be tested on large animals, which are similar with respect to the human functional-anatomical scale to further translate these results to clinic trials. In this and our recent study, a comparative analysis of combined EES and triple gene treatment in two animal models, rats and pigs with SCI, revealed some common patterns in post-traumatic molecular and cellular changes during brain remodeling, which correspond to the improvement in locomotor outcome. However, the functional recovery in pigs 2 months after SCI corresponded to the level of recovery in rats after 1 month of spinal cord contusion injury. The small size, high level of metabolism, and different regeneration capacities in rodents likely form a better functional outcome when compared to large animals. Although we have confirmed the positive effect of combined EES and ex vivo triple gene therapy in pigs with SCI, we believe that our results should be considered preliminary due to the need for further investigation on a comparative study of animals subjected to EES or gene therapy alone.

## 4. Conclusions

This study, for the first time, employs a combination of EES-facilitated locomotor training with intrathecal administration of transgenes encoding neuroprotective molecules (VEGF, GDNF, and NCAM), using UCBC as a delivery system for the treatment of SCI in large animals. These preliminary results indicate the need for further in-depth, discrete analysis of EES and ex vivo triple gene therapy impact in a combined treatment of SCI.

## 5. Material and Methods

### 5.1. Animals and Care

Six female adult Vietnamese Pot-Bellied pigs (20–25 kg) were used for this study. Animals were obtained from the Kazan State Academy of Veterinary Medicine by N.E. Bauman (Kazan, Russia) and were kept one per a housing area for 2 weeks before the surgery. Throughout the investigation, pigs were kept in a 12 h light/dark regimen at a temperature of 24–25 °C with controlled air conditioning and properly organized feeding. All experiments were conducted according to the animal protocol approved by the Kazan State Medical University Animal Care and Use Committee.

### 5.2. Experimental Groups

In the study, animals were divided in three experimental groups: Intact (I, *n* = 2), control (C, *n* = 2), and therapeutic group with combined EES and gene therapy (GT-ES, *n* = 2). Animals from control and therapeutic groups were implanted with electrodes and received SCI. Control pigs, 4 h after SCI, received an intrathecal injection of 200 μL 0.9% NaCl. Pigs from the therapeutic group received 2 × 10^6^ of gene-modified UCBC in 200 μL of 0.9% NaCl and 2 weeks after SCI underwent EES accompanied with training on a treadmill. Intact healthy pigs were used to collect basic behavioral, electrophysiological, and histological data for comparative analysis. Here, we did not use a sham group. We employed the established model of SCI in pigs [45] and it was shown that the morphological characteristics of the spinal cord [77] and the PTIBS score [78] in sham pigs do not differ from intact animals. The small animal number in this exploratory study, similar to other pre-clinical studies performed on a small number of large animals [79,80], was explained by the difficult maintenance and care of large animals with SCI. Moreover, there are case reports in the application of EES in one patient with paraplegia [34,35]. We did not perform any hypothesis testing and estimation procedures using collected data due to the limited sample size consideration. Average values presented in the article are intended for descriptive purposes only.

### 5.3. Gene-Modified Human Umbilical Cord Blood Mononuclear Cells

In the study for ex vivo gene therapy, the umbilical cord blood mononuclear cells (UCBC) transduced with adenoviral vectors carrying recombinant human genes *vegf165, gdnf,* and *ncam1* were employed. The umbilical cord blood was collected after obtaining an informed consent of the pregnant and prenatal screening for contraindications to the blood donation. All manipulations with the blood were conducted in strict compliance with the guidelines established by the Stem cell bank of Kazan State Medical University. Preparation of the gene-modified UCBC was performed according to the previously described protocol [30]. Viral vectors were prepared based on the human adenovirus serotype 5 (Ad) in the N. F. Gamaleya Federal Research Center for Epidemiology and Microbiology (Moscow, Russia), as described previously [31]. The titers of Ad5-VEGF (2.1 × 10^9^ PFU/mL), Ad5-GDNF (3.4 × 10^9^ PFU/mL), and Ad5-NCAM (2.0 × 10^9^ PFU/mL) were determined by the plaque formation technique in the HEK-293 cell culture. UCBCs isolated by the Ficoll density gradient separation were simultaneously transduced with adenoviral vectors in an equal ratio of each vector including Ad5-VEGF (1/3), Ad5-GDNF (1/3), and Ad5-NCAM (1/3) with a multiplicity of infection of 10 (MOI = 10). Transduced cells were incubated for 14 h. For a single injection, 2 × 10^6^ UCBC + Ad5-VEGF + Ad5-GDNF + Ad5-NCAM in 200 μL of saline was prepared. The analysis of recombinant mRNA synthesis was performed 72 h after culturing of the transduced UCBC by the RT-PCR assay.

### 5.4. Electrode Implantation

Stimulating electrodes were implanted at the C5 cervical and L2 lumbar spinal segments. EES below the injury (L2) was utilized to activate spinal neural networks [36,37] and facilitate the motor performance after SCI during training on a treadmill. A cervical spine (C5) stimulation has been applied in this study to engage cervical networks responsible for forelimb functions and to facilitate propriospinal multisegmental coordination [81,82]. EES above the injury may also promote regrowth of the axons through the lesion site [41,51]. All animals were implanted with stimulating and reference teflon-coated wire electrodes (AS632, Cooner Wire Company, Chatsworth, CA, USA) and connected to the 12-channel connector (Omnetics Connector Corporation, Minneapolis, MN, USA). Animals were anesthetized with an intramuscular injection in the back of the neck with 10 mg/kg of Zoletil 100 (Virbac Laboratoires, Carros, France) and 40 mg/kg of Xyla (Interchemie werken “De Adelaar” B.V., Castenray, Netherlands), and subsequently connected to an inhalation anesthesia apparatus (Minor Vet Optima, Zoomed, Moscow, Russia), through which isoflurane (Laboratorios Karizoo, S.A., Barcelona, Spain) was administered in a mixture with oxygen. After a laminectomy of the C4 and C5 vertebrae, the upper stimulating electrode was inserted through the window in the yellow ligament between C3 and C4 and pulled into the region of the C5 arch and tied to the dura matter, using polypropylene suture USP 7-0. The reference electrode was implanted intramuscularly into the neck muscles. Afterwards, the wire was tunneled from C5 to the skin incision at T14-L3, through which the reference and second stimulating electrodes were pulled to the lumbar region. The second stimulating electrode was fixed to the dura matter with a suture at L2 and the reference electrode was fixed subfascial in the abdomen region. To install the 12-channel connector, a small skin incision on a withers site lateral from the midline was performed and a connector was inserted through the skin incision and sutured tightly (Figure 1).

### 5.5. SCI Model

A moderate contusion injury of the spinal cord was performed 2 weeks after implantation of the electrodes, as described previously [17]. In short, pigs under deep anesthesia induced with Zoletil 100 and Xyla and maintained with 2.5% isoflurane in a mixture with oxygen underwent a laminectomy at the T8–T9 vertebral level. The 50 g metal rod with a diameter of 9 mm of the custom-made weight-drop device was centered above the exposed dura matter. The contusion injury was caused by the metal rod falling from a height of 50 cm. A single hit to the spinal cord was performed while the animal’s spine was fixed in an optimal position with an elongated straight spine position, followed by the hind limb skeletal muscles contraction. Before closing the wound, two surgeons confirmed the signs of the contusion injury according to the appearance of the consistent hematoma at the site exposed to the metal rod. The final verification of the spinal contusion severity in all experimental animals was obtained in the histological study of the nervous tissue sparing. A silicone 18 Fr urinary Foley catheter (“Vogt medical vertieb GMBH”, Karlsruhe, Germany) was placed during the surgery and was maintained post-operatively. 

### 5.6. Gene Therapy

Four hours after SCI, pigs received ex vivo gene therapy (Figure 1). Supplemental doses of Zoletil were administered i.p. as needed to keep animals in deep anesthesia and their body temperature during this period was maintained at 38 °C with a heating pad. After a laminectomy at the L4-L5 level, pigs were subjected to an intrathecal injection of 2 × 10^6^ gene-modified UCBC (UCBC + Ad5-VEGF + Ad5-GDNF + Ad5-NCAM) in 200 μL of saline using a U-100 insulin syringe and a 26 gauge needle under the microscope MC-2-ZOOM (Ningbo Sheng Heng Optics & Electronics Co. Ltd., Ningbo, China).

### 5.7. Post-Operative Care

After SCI, all animals received post-operative care to maintain a stable state of health and avoid post-operative complications with adequate analgesia and antibacterial therapy. Along with medications, the vital signs (e.g., respiratory rate, body temperature, post-operative wound condition, etc.) were assessed daily. Other metrics, such as water and feed intake, excretion of urine and feces, movement activity and signs of pain syndrome (vocalization, depressed behavior, and jitteriness), were also assessed and registered throughout the whole 60 days of the experiment.

### 5.8. Epidural Electrical Stimulation

Epidural electrical stimulation (EES) was performed with Digitimer DS7A (Digitimer Ltd., Welwyn Garden, UK) and stimulation parameters (25–35 Hz with 7‒25 mA and 0.2 ms pulse duration) were configurated. Data were recorded using the LabChart data acquisition and system (AD Instruments Inc., Colorado Springs, CO, USA). EES started 2 weeks after the SCI injury and was applied every 2nd day for 30 min in the morning (above the lesion site, at C5) and in the evening (below the injury, at L2) with a usual interval of 7 h between both sessions. This regimen was chosen to give the animals rest between both procedures. The morning session included stimulation at the C5 level (for stimulation of the cervical network related to forelimb functions and a potential neuroregenerative effect), with a current intensity selected individually for each animal, which was usually in the range of 7–15 mA. The evening session consisted of stimulation below the injury at L2 (for activation of the central pattern generators in order to facilitate the motor performance during training on a treadmill) with a current intensity in the range of 13–25 mA. EES at the lumbar level was combined with training on a treadmill by Torneo T-530 Olympia (Zhejiang kingdom sports CO., LTD, Huzhou City, Zhejiang Province, China). During EES, pigs were secured in a body weight support system so that the animals remained within the treadmill and the load on the hind limbs was from 5% to 20% of their weight. A successful EES triggered hind limb stepping was matched with the treadmill belt speed (0.3–0.4 m/s).

### 5.9. Porcine Thoracic Injury Behavioral Scale

Recovery of the locomotor function was evaluated using the porcine thoracic injury behavior scale (PTIBS) assessment [45]. PTIBS was determined once at 5, 6, 7, and 8 weeks after SCI. The locomotor activity of hind limbs was estimated by two observers in a blinded manner with respect to the experimental groups, following the 10-point scale when each animal was placed in the center of the arena (5 × 5 m) for walking.

### 5.10. Hind Limb Joints Kinematics

Joint kinematics were evaluated by changes in angular degrees in the hip, knee, and ankle joints of the hind limbs of pigs, while walking on a treadmill. Two weeks before the implantation of the electrodes and 1 week after, animals were trained to walk on a treadmill. During the training period, pigs were fixed as described above. The training was performed with a treadmill speed of 0.3–0.4 m/s. Hind limb joints kinematics were assessed 1 week before SCI to collect basic data and at 5, 6, 7, and 8 weeks after SCI. In the projections of the ileal crest, the greater trochanter of the femur, the knee, ankle joints, and the hoof of the left hind limb, color marks were applied. A video recording of the color marks during pigs walking on a treadmill was carried out using a Canon Power Shot S5 IS camera (Tokyo, Japan). A video analysis of the hip, knee, and ankle joints kinematics was performed using the Kinovea 0.8.25 software [83]. The joint movement volume was calculated as the difference between the maximum and minimum joint angles when evaluating five step cycles.

### 5.11. Electrophysiological Study

The electromyography (EMG) of the soleus muscle evoked by electrical stimulations of the sciatic nerve was performed 60 days after SCI. EMG was carried out under deep anesthesia induced with Zoletil 100 and Xyla and maintained with 2.5% isoflurane in a mixture with oxygen. In the projection of the left sciatic nerve (2 cm below the greater trochanter of the femur and 1 cm down from the femur), two needle stimulating electrodes were implanted. The recording needle electrodes were implanted into the m. soleus at the site between the medial and lateral gastrocnemius muscle. Stimulation was performed by single rectangular pulses with a frequency of 0.6 Hz, a pulse duration of 0.2 ms, and a current of 4–72 mA using Digitimer DS7A (Digitimer Ltd., Welwyn Garden, UK). Biopotentials were enhanced with an amplifier (Biosignal amplifier, g.tecmedical engineering GmbH, Schieldberg, Austria) and analyzed using the LabChart data acquisition and analysis system (AD Instruments Inc., Colorado Springs, CO, USA). The H-reflex from spinal motoneurons and the M-response from the soleus muscle were measured.

### 5.12. Samples Collection

Experimental animals were sacrificed 60 days after SCI. Spinal cords along the site of the injury were harvested together with the vertebrae. The obtained caudal part of the spinal cord below the injury was divided into three caudal segments (CS1, CS2, and CS3) (Figure 1). The samples from CS1 and CS2 were processed for a morphological and immunofluorescent study and from CS3 for a molecular analysis. To assess the effect of the therapeutic approach, in this study, each of the caudally located areas was analyzed separately to clarify the quality of regeneration. An immunofluorescent examination of the CS2 region was performed to address a question of the preservation of cells, the state of macro-and microglia, chlorine homeostasis, and synaptic communications. The study of the state of the neuromediator and cytokine systems by molecular methods in the CS3 area was performed as an independent task to assess the state of excitatory and inhibitory components of the motor circuitry. The inflammatory response was assessed with a multiplex cytokine analysis. The CS1 and CS2 segments were fixed in 4% paraformaldehyde in phosphate-buffered saline (PBS, pH 7.4) at 4 °C. The CS3 segments were frozen in liquid nitrogen and kept at −80 °C. Skeletal tibialis anterior muscles from both hind limbs were harvested and immediately weighed. The fragments from the middle part of the muscles were fixed in 4% paraformaldehyde in PBS at 4 °C for a morphological study.

### 5.13. Morphometric Analysis

Total cross sections of spinal cords from CS1 and CS2 segments with a thickness of 20 μm were obtained using a cryostat (Microm HM 560, Thermo Scientific, Waltham, MA, USA). Frozen sections were mounted on slides and stained with hematoxylin and eosin. Digitized images of the spinal cord captured with a 4-fold magnification of the microscope were analyzed using the ImageJ (NIH) software. The volume of pathological cavities and the preservation of gray matter relative to the entire area of the gray matter of the spinal cord were measured. Frozen 10 μm cross sections of tibialis anterior muscles were mounted on slides and stained with hematoxylin and eosin. Digitized images of the skeletal muscles were captured with a 20-fold magnification of the microscope and analyzed using the ImageJ (NIH) software. The area of 100 muscle fibers in the left and 100 muscle fibers in the right hind limbs were measured. Then, the average values were calculated for both animals in the group.

### 5.14. Immunofluorescence

Immunofluorescent staining was performed on free-floating cross sections (20 μm) obtained from CS2 segments (Figure 1). Immunoexpression of target protein markers in neural and glial cells were revealed using specific primary antibodies (Ab). Responses of glial cells were assessed with Ab to glial fibrillary acidic protein (GFAP) for astrocytes, Ab to oligodendrocyte transcription factor (Olig2) for oligodendroglial cells, and Ab to ionized calcium binding adaptor molecule 1 (Iba1) for microglia (Table 1). Abs to Hsp27 and Caspase3 were employed to evaluate the survivability of spinal cord cells. The functional recovery on neural cells was analyzed with Abs against potassium-chloride cotransporter protein (KCC2), Synaptophysin, and postsynaptic density protein of 95 kDa (PSD95). For immunofluorescent labeling, sections were incubated with appropriate secondary antibodies (Table 1). Nuclear counterstaining was performed with DAPI (10 μg/mL in PBS). Immunofluorescence images were taken by the LEICA TCS SP5 MP microscope (Leica Microsystems; Wetzlar, Germany) using identical confocal settings. The pattern of target proteins immunoexpression was evaluated in ventral horns of spinal cords with an area of 0.05 mm^2^ using the ImageJ (NIH) software. The number of immunopositive cells expressing Caspase3, Hsp27, GFAP, Olig2, and Iba1 was counted in regard to the nuclear counterstaining with DAPI. The level of synaptic proteins (Synaptophysin and PSD95) and KCC2 immunoexpression in spinal cord sections was evaluated as mean pixel intensities using the ImageJ software (NIH). Average values of the obtained measurements were calculated for each animal.

### 5.15. RT-PCR Assay

The expression of genes encoding postsynaptic proteins in CS3 segments was studied using the real-time polymerase chain reaction (RT-PCR). Total RNA was isolated using the RNeasy Mini Kit (Qiagen) in accordance with the manufacturer’s instructions. The cDNA synthesis was performed using random hexamers and RevertAid Reverse Transcriptase (Thermo Fisher Scientific). Quantification of the genes expression was carried out using the CFX96 thermal cycler (BioRad). The reaction mixture included qPCRmix-HS SYBR (Eurogen), cDNA samples, specific primers for the postsynaptic genes of the following neurotransmission systems: Cholinergic (*Chrm1*); glutamatergic (*Grin2a*); glycinergic (*Glra1*); and GABAergic (*Gabra2*) (Table 2). Expression levels of the *Gapdh* gene were used for normalization. Each reaction was carried out in two technical repeats. The *ΔΔCt* (Livak) method was used to calculate the average relative target genes expression normalized by *Gapdh*.

### 5.16. Multiplex Cytokine Analysis

A simultaneous analysis of the level of 13 cytokines/chemokines was determined in the samples of the spinal cord of intact and experimental animals at 60 days after SCI. Freshly frozen spinal cord samples from CS3 segments were ground in a cold RIPA Lysis Buffer (150 mM NaCl, 5 mM EDTA, 50 mM Tris, 1.0% NP-40, 0.5% sodium deoxycholate, 0.1% SDS) with Protease Inhibitor Cocktail (Sigma-Aldrich, P2714) and 1 mM PMSF, using a FastPrep-24 homogenizer. The prepared lysates were centrifuged at 14,000× *g* for 10 min at 4 °C and the obtained supernatants were transferred into new tubes, frozen, and stored at −80 °C until use. The multiplex analysis was performed using the Porcine Cytokine/Chemokine MILLIPLEX^®^ magnetic bead-based multi-analyte panel containing the following target analytes: GM-CSF, IL-1ra, IL-2, IL-6, IL-10, IL-12, IL-18, TNFα, IFNγ, IL-1β, IL-1α, IL-4, and IL-8 (Millipore). The data were analyzed with a MAGPIX^®^ system and the Analyst 5.1 software (Merck). All values were log-transformed and presented as a heatmap with hierarchically clustered proteins.

## Figures and Tables

**Table 1:** Primary and secondary antibodies for immunofluorescence.

Antibody	Host	Dilution	Source
Caspase3	Rabbit	1:200	Abcam (Cat # ab13847)
GFAP	Mouse	1:200	Santa Cruz (Cat # sc-33673)
Hsp27	Rabbit	1:200	Abcam (Cat # ab12351)
Iba1	Rabbit	1:150	Abcam (Cat # ab178847)
KCC2	Rabbit	1:200	Abcam (Cat # ab49917)
Olig2	Rabbit	1:100	Abcam (Cat # ab109186)
PSD95	Rabbit	1:200	Abcam (Cat # ab18258)
Synaptophysin	Rabbit	1:200	Abcam (Cat # ab32127)
Anti-rabbit IgG conjugated with Alexa 488	Donkey	1:200	Invitrogen (Cat # A-21206)
Anti-mouse IgG conjugated with Alexa 488	Donkey	1:200	Invitrogen (Cat # A-21202)
Anti-rabbit IgG conjugated with Alexa 647	Donkey	1:200	Invitrogen (Cat # A-31573)
Anti-rabbit IgG (Texas Red) pre-adsorbed	Donkey	1:200	Abcam (Cat # ab7081)

**Table 2:** Nucleotide sequences of the primers and products of real-time PCR.

Genes and Nucleotide Sequences	Product Length, bp	GC, %	Tm, °C
*Chrm1* (NM_214034)	260		
F: GAAAAGCTTGGCTCAGAGGGA		52.38	60.27
R: ATGACATAGTGGGACCGTCG		55.00	59.26
*Grin2a* (XM_021086653)	219		
F: TGTTGGAGGTCCAATAGTGCC		52.38	60.00
R: TTGCCAACATACCTAGGGGG		55.00	59.08
*Glra1* (XM_013984909)	170		
F: GTGTGCAATCCCCAATGCAG		55.00	60.11
R: GCAGCCTACGGACTCACATT		55.00	60.11
*Gabra2* (XM_013978645)	244		
F: CACGCCAGAACCCAACAAGA		55.00	60.82
R: GTACATGGCAAAACAAACCAGG		45.45	58.61

## Supplementary Materials

The following are available online at https://www.mdpi.com/1422-0067/21/23/8896/s1, Video S1: Representative mini pig before injury and after spinal contusion during training on a treadmill at 2, 4, and 8 weeks post injury without and with EES applied at L2 lumbar segment. Animal demonstrate gradual improvement in motor performance in presence of L2 electrical epidural stimulation used for EES-enabled training.
